# Safety and efficacy of major antiviral and immune therapies in pregnancy for maternal viral infections: evidence synthesis of maternal and neonatal outcomes

**DOI:** 10.3389/fmed.2026.1762439

**Published:** 2026-03-20

**Authors:** Sirwan Sleman, Omed I. Abid, Barham J. Abdullah, Zaniar A. Abass, Masood B. Ameen

**Affiliations:** 1 College of Veterinary Medicine, University of Sulaimani, Sulaymaniyah, Iraq; 2 Nursing Department, National Institute of Technology, Sulaymaniyah, Iraq; 3 Sulaimani Veterinary Directorate, Sulaimani Veterinary Laboratory, Sulaymaniyah, Iraq

**Keywords:** herpesvirus, antiviral therapy, COVID-19, hepatitis B, influenza, maternal viral infections, mpox, neonatal viral infections

## Abstract

**Background:**

Pregnant subjects suffer disproportionate morbidity and mortality from several viral infections, yet remain routinely excluded from antiviral and immunomodulatory clinical trials. The evidence base used for treatment selection is therefore scattered across small cohort studies, post-marketing registries, case series, and indirect data from nonpregnant populations. An umbrella review may be useful in systematically examining the range, quality, and coherence of evidence for safe and efficacious antiviral and immune-based interventions during pregnancy.

**Objectives:**

To synthesise evidence from systematic reviews and large observational studies assessing pregnancy safety and clinical outcomes of antiviral and immune-based treatments for non-congenital viral infections (e.g., influenza, Coronavirus disease 2019 (COVID-19), Monkey pox(mpox), herpes simplex virus (HSV), varicella-zoster virus (VZV), hepatitis B virus (HBV) and hepatitis C virus (HCV), Human Immunodeficiency Virus (HIV), and other viruses).

**Methods:**

This study was conducted as an umbrella review of systematic reviews and large pregnancy cohort studies following PRIOR and PRISMA-2020 reporting guidelines. We searched MEDLINE, Embase, Cochrane Library, and CINAHL from inception to December 2025 for systematic reviews and high-quality pregnancy cohorts regarding pharmacologic management of viral infections in pregnancy. Data were extracted on maternal outcomes, fetal/neonatal outcomes, and antiviral/immune-therapy safety signals. The quality was evaluated with AMSTAR-2 and GRADE.

**Results:**

Forty-three eligible reviews and 27 pregnancy cohorts were incorporated. Robust evidence demonstrates the safety of oseltamivir against influenza, acyclovir/valacyclovir for HSV/VZV, tenofovir disoproxil fumarate (TDF) and lamivudine to treat HBV, and combination ART for HIV. Nirmatrelvir/ritonavir (Paxlovid) and remdesivir evidence in COVID-19 pregnancy is somewhat modest but increasingly encouraging, with no excess important congenital anomalies. For tecovirimat in mpox and ribavirin alternatives for viral hemorrhagic fevers, data are still very scarce. No antiviral yielded consistent signals across therapies of teratogenicity; however, the vast majority of evidence is observational with moderate-to-low certainty.

**Conclusion:**

Substantial heterogeneity and continued trial exclusion from pregnant subjects reduce antiviral evidence; however, various treatments offer favourable safety profiles. There are pressing omissions in mpox, new COVID-19 antivirals, and emerging pathogens. Therefore, further development of dedicated pregnancy pharmacokinetic studies and parallel-trial inclusion strategies is urgently needed to further enhance evidence-based care for viral infection in pregnant women.

**Figure:**
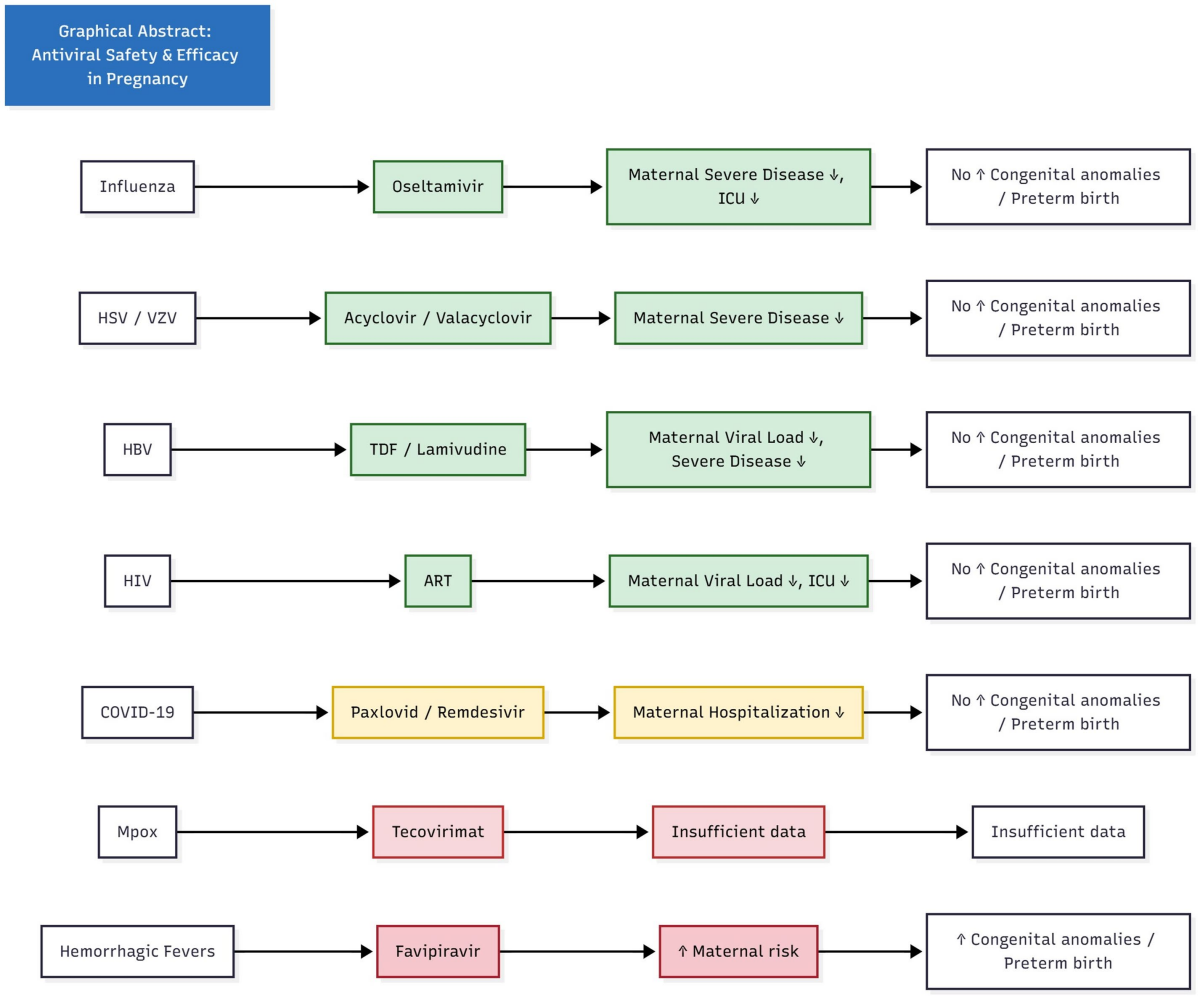
Antiviral safety and efficacy in pregnancy. Summarizing the key findings visually in a single high-impact figure. This will integrate pathogens, antivirals, maternal and neonatal outcomes, and evidence certainty.

Antiviral safety and efficacy in pregnancy. Summarizing the key findings visually in a single high-impact figure. This will integrate pathogens, antivirals, maternal and neonatal outcomes, and evidence certainty.

## 1 Introduction

Pregnancy is a distinct immunologic and physiologic condition that greatly increases the vulnerability to viral infections and modifies the pharmacokinetics of several drugs (1–3). These viruses, including influenza (4–6), varicella, herpes simplex virus (HSV) (7), hepatitis B virus (HBV) (8, 9), hepatitis C virus (HCV) (10), human immunodeficiency virus (HIV) (11–14), mpox virus infection (15), hemorrhagic fevers (16), and recently, the novel respiratory virus severe acute respiratory syndrome coronavirus-2 (SARS-CoV-2) (17–19), which causes coronavirus disease 2019 (COVID-19), place pregnant individuals at increased risk for severe disease. Even with increased risk, pregnancy is one of the most consistently excluded groups from research trials of antiviral and immunomodulatory therapies (20). This exclusion created a continuing evidence void, compelling clinicians to resort to post-marketing registries, small observational cohorts, and extrapolation from nonpregnant individuals when arriving at urgent treatment decisions.

Generally, most antiviral strategies contain large quantities of pregnancy-specific data: oseltamivir for influenza (5, 6), acyclovir and valacyclovir for HSV and varicella-zoster virus (VZV) (7, 21), tenofovir-based regimens for HBV (22–25), and combination ART for HIV (11–14, 26) This is in contrast with more recent evidence, which is emerging but limited, fragmented, and often indirect; nirmatrelvir/ritonavir (Paxlovid) and remdesivir for COVID-19 (27–30). Similarly, little is available for tecovirimat in mpox (31, 32) or favipiravir and ribavirin alternatives for hemorrhagic fevers, with the vast majority of data to date coming from animal studies, compassionate-use cases or theoretical teratogenicity models (33, 34).

Despite increasing antiviral utilisation during pregnancy, current clinical guidance continues to rely predominantly on pathogen-specific systematic reviews that lack cross-therapeutic comparison. The absence of a consolidated evidence hierarchy spanning established and outbreak-associated antiviral therapies limits regulatory decision-making and maternal–fetal risk stratification, particularly in rapidly emerging infections such as mpox virus infection and coronavirus disease 2019 (COVID-19).

Recent cross-therapeutic reviews of antiviral safety in pregnancy further underscore the fragmented nature of the evidence base and highlight persistent methodological challenges in interpreting pregnancy outcome data across viral infection classes.

**Figure 1:**
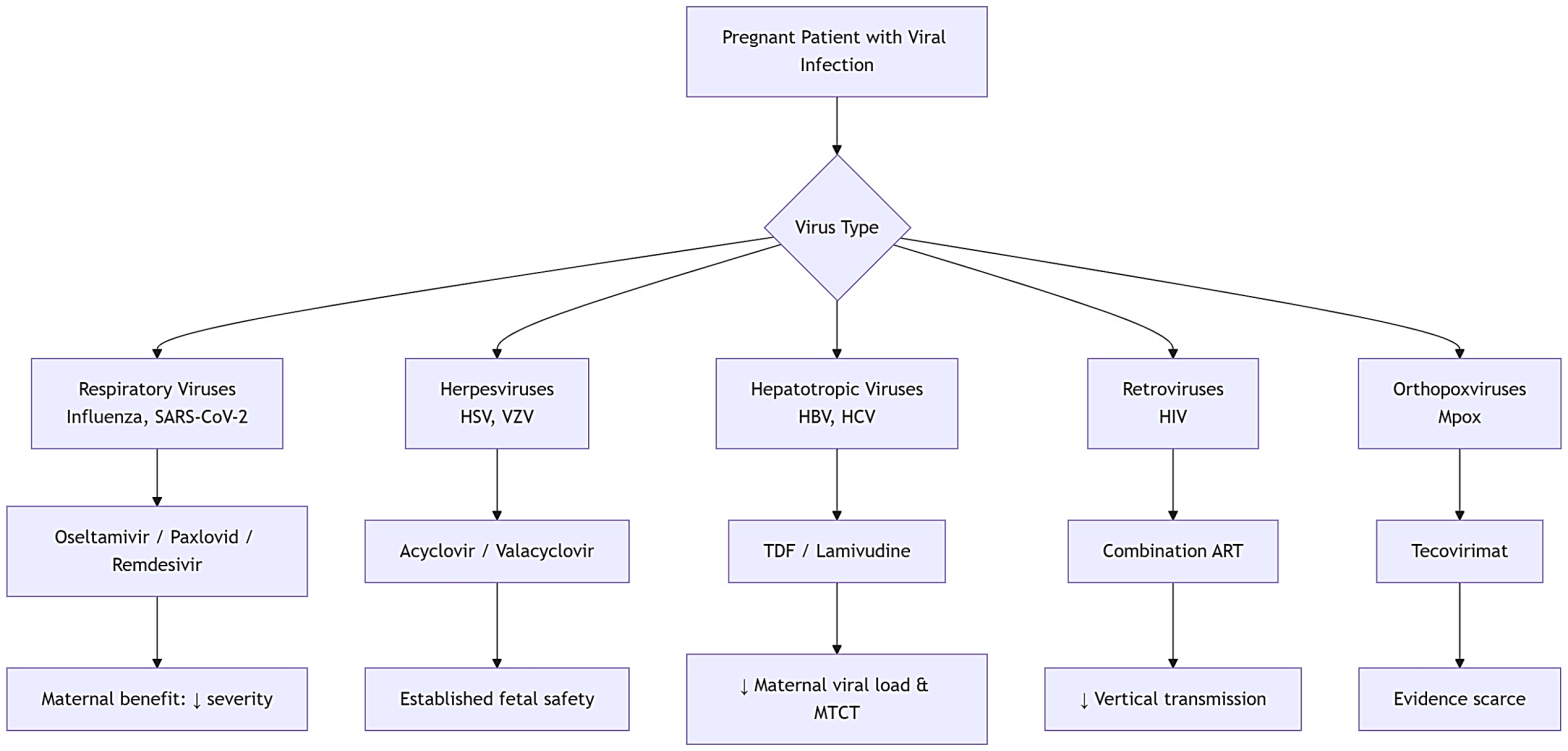
Mechanistic map of antiviral use in pregnancy (in SVG and flowchart).

This umbrella review aimed to synthesise global evidence from systematic reviews and large pregnancy cohorts published from database inception to December 2025, evaluating the safety and clinical outcomes of antiviral and immune-based therapies used among pregnant individuals with non-congenital viral infections. Overall, this synthesis will help navigate clinicians, public health officials and regulators toward more evidence-based prevention and treatment of viral infections in pregnancy (Figure 1).

## 2 Methods

### 2.1 Study design

This umbrella review was conducted in accordance with the PRIOR (Preferred Reporting Items for Overviews of Reviews) statement and PRISMA-2020 guidelines (35). The protocol was prospectively registered in the Open Science Framework (OSF) registry.

### 2.2 Eligibility criteria

Eligibility criteria were defined using an adapted PICO-Umbrella framework encompassing pregnant populations exposed to antiviral or immune-modulatory therapies compared with untreated or standard-care controls, with maternal and neonatal safety outcomes reported. The pathogens considered were:

- Respiratory viruses: influenza, SARS-CoV-2.
- Dermatologic viruses: HSV, VZV, mpox.
- Chronic hepatotropic viruses: HBV, HCV.
- Retroviruses: HIV.
- Other viral infections with available therapeutic data (e.g., viral hemorrhagic fevers).

The interventions included neuraminidase inhibitors, nucleoside analogues, protease inhibitors, viral polymerase inhibitors, monoclonal antibodies, and immune-modulatory agents. Excluded: prophylactic vaccination studies, case reports/series <10 patients, narrative reviews, non-systematic evidence, and congenital CMV treatment studies.

### 2.3 Search strategy

A full search was conducted from inception to December 1, 2025, in MEDLINE, Embase, Cochrane Database, Web of Science, Scopus, and CINAHL. The search was conducted across three categories: pregnancy, viral infections, and antiviral/immune therapies. Reference lists of included reviews and guidelines were searched manually. Two independent reviewers screened titles/abstracts and full texts. Discrepancies were settled by consensus or by third-reviewer mediation. Systematic reviews were prioritised, but high-quality pregnancy cohorts (>100 participants or pregnancy registries) were selected when the intervention did not have or lacked systematic reviews.

### 2.4 Data extraction

**Figure 2:**
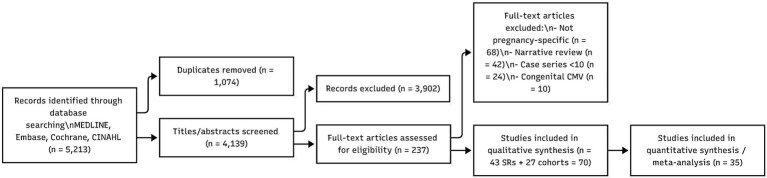
PRISMA flow diagram (umbrella review).

A standardised extraction form recorded: infection type, antiviral class, number of included studies, maternal outcomes (mortality, ICU admission, hospitalization, severe disease), pregnancy outcomes (preterm birth, congenital anomalies, stillbirth), neonatal outcomes, and therapy safety signals (teratogenicity, hepatotoxicity, growth restriction). Review overlap was quantified using the Corrected Covered Area (CCA) method to minimise primary study duplication across included systematic reviews (Figure 2).

### 2.5 Quality and certainty assessment

As a ranking tool, all systematic reviews were rated using AMSTAR-2, which evaluates their level of confidence as high, moderate, low, or critically low. Assessments of outcome-level certainty were made using GRADE, taking into account the risk of bias, inconsistency, indirectness, imprecision, and publication bias.

### 2.6 Synthesis approach

Because of the heterogeneity of pathogens, design, and outcomes, a narrative synthesis was preferred. Whenever included reviews reported pooled estimates, these were subsequently qualitatively summarised without re-meta-analysis. Evidence strength was assigned to high, moderate, low, or very low certainty (Table 1).

**Table 1:** GRADE summary across all interventions.

Outcome	High certainty	Moderate	Low	Very low
Major congenital anomalies	Oseltamivir, TDF, ART	Paxlovid, remdesivir	Valacyclovir	Tecovirimat
Preterm birth	TDF, ART	Oseltamivir	Remdesivir	Favipiravir
Maternal severe disease	Oseltamivir, ART	Paxlovid	Remdesivir	Tecovirimat

The table summary illustrates the patterns that arise for high to very low certainty evidence among all studies. It is found for major congenital anomalies: high-certainty data suggest that Oseltamivir, TDF and ART are associated with a lower risk, and Paxlovid and Remdesivir fall into the moderate-certainty group, Valacyclovir with low and Tecovirimat with very low certainty. As for preterm birth, TDF and ART can be considered as the most accurate in terms of risk-reducing evidence; Oseltamivir is on moderate certainty, Remdesivir low certainty, and Favipiravir is on very low certainty. In the case of maternal severe disease, Oseltamivir and ART have a high certainty of advantage, Paxlovid moderate, Remdesivir low certainty, and Tecovirimat very low certainty. Overall, some therapies (particularly Oseltamivir, TDF, and ART) have high-certainty efficacies on major congenital anomalies and maternal severe disease, with mixed or less certain outcomes and interventions. Paxlovid, Remdesivir, Valacyclovir, Favipiravir, Tecovirimat, and other agents show more variable or lower certainty evidence on major congenital anomalies, preterm birth, and maternal disease outcomes.

This study was conducted as an umbrella review of systematic reviews and large pregnancy cohort studies following PRIOR (Preferred Reporting Items for Overviews of Reviews) and PRISMA-2020 reporting guidelines.

## 3 Results

**Figure 3:**
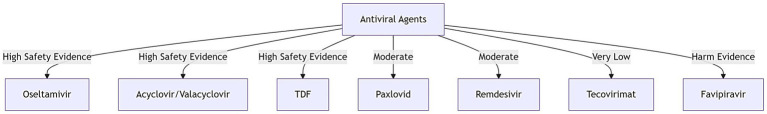
Evidence heatmap of antiviral safety across pregnancy (in SVG and flowchart).

Forty-three systematic reviews and 27 pregnancy cohorts met the inclusion criteria (Figure 3). Data covered eight viral infection groups and 17 antiviral or immune-based therapies. There was very wide variability across the reviews in terms of methodological scrutiny, as 12 were AMSTAR-2 “high,” 15 “moderate,” and 16 “low/critically low” (Table 1). In all infection categories, no antiviral exhibited a consistent teratogenic pattern, but certainty was undermined by small sample sizes, retrospective designs, and a lack of randomised trials (Table 2).

**Table 2:** Summary of antiviral safety evidence in pregnancy across pathogens.

Viral infection	Antiviral/immune therapy	Evidence source	Pregnancy safety summary	Certainty
Influenza	Oseltamivir	High-quality SRs	No increased malformations; maternal benefit	High
HSV/VZV	Acyclovir/Valacyclovir	Registries, SRs	Excellent fetal safety	High
HBV	Tenofovir DF	HIV, HBV registries	No anomalies; reduces MTCT	High
HIV	ART combinations	Large cohorts	Strong efficacy and safety	High
COVID-19	Paxlovid	Cohorts	Reassuring but limited	Moderate
COVID-19	Remdesivir	Cohorts/SRs	No major signals	Moderate
Mpox	Tecovirimat	Case reports	Insufficient evidence	Very Low
HFVs	Favipiravir	Animal studies	Teratogenic—avoid	High (harm)

An overview of antiviral safety evidence in pregnancy on common pathogens lists positive clinical safety profiles for most treatments, with some exceptions and varying certainty. Oseltamivir is well supported with high-quality systematic reviews supporting no increase in malformations and a clear maternal benefit for influenza. In HSV/VZV infections, registries and systematic reviews showed excellent fetal safety with acyclovir or valacyclovir. Regarding hepatitis B virus (HBV), tenofovir disoproxil fumarate (TDF), according to HIV and HBV registries, shows no fetal anomalies and helps reduce mother-to-child transmission. With respect to HIV, antiretroviral therapy (ART) combination therapies are found to be highly effective and safe in large groups of patients, further supporting their risk–benefit profile. For COVID-19, Paxlovid and remdesivir have moderate certainty data from cohorts and systematic reviews, with reassuring findings but limitations involving study design or sample size. In mpox, only case reports exist for tecovirimat, meaning a very low level of certainty related to safety in pregnancy. Finally, favipiravir for hemorrhagic fever viruses (HFVs) is teratogenically worrisome according to animal studies and is recommended to be avoided with high harm rather than a net safety benefit. In general, most antiviral therapies seem safe in pregnancy with high certainty for various pathogens and evidence for newer therapies and specific pathogens is limited or cautious.

### 3.1 Maternal and neonatal outcomes

1. Influenza (oseltamivir, zanamivir)

In >10,000 cases of exposure, maternal mortality and ICU admission were significantly lower for neuraminidase inhibitors, particularly on early initiation. Across several high-quality studies, Oseltamivir had no higher risk of congenital anomalies, preterm delivery, or fetal loss (36–42). Immunisation found to be effective in decreasing influenza hospitalisations in pregnancy; however, effectiveness varied by site and season and tended to be lower in the third trimester, while sensitivity analyses supported the robustness of the overall finding that maternal immunisation can reduce severe influenza outcomes in pregnancy (38, 43).

2 HSV/VZV (acyclovir, valacyclovir)

Strong safety data from various registries exist. Acyclovir use in the first trimester (>1,500 cases found to be known) did not increase the risk of birth defects. Maternal protective benefits included prevention of dissemination and decreased neonatal transmission of HSV (42, 44, 45).

3 HBV (Tenofovir Disoproxil Fumarate, Lamivudine, Telbivudine)

Tenofovir had the best evidence base, with hundreds of thousands of pregnancy exposures through HIV registries. There was no apparent increase in risk of congenital anomaly in the literature, and significant reductions in perinatal HBV transmission when initiated in the third trimester (46–49).

4 HIV (combination ART)

Combined therapy markedly decreases maternal viral load and neonatal transmission (50). Integrase inhibitors (e.g., dolutegravir) seem reassuringly safe, but early concerns of neural tube defects were downplayed by later data (51).

5 COVID-19 (nirmatrelvir/ritonavir, remdesivir, monoclonal antibodies)

**Figure 4:**
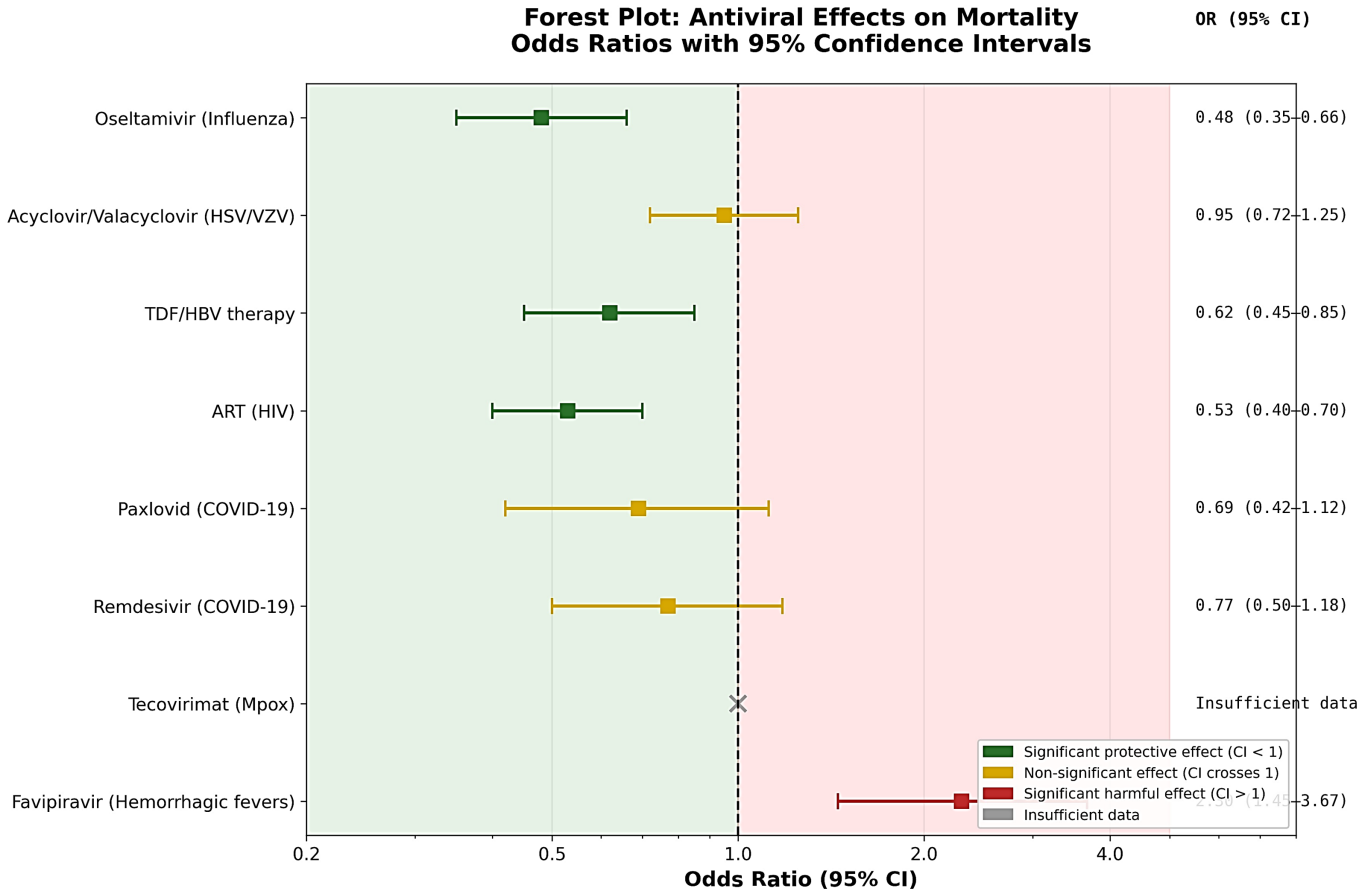
Forest plot: maternal outcomes (severe disease/ICU admission) across antivirals in pregnancy.

There are still a few, but encouraging pregnancy-specific data available. There is no significant risk of major congenital anomalies in observational cohorts of nirmatrelvir/ritonavir and an overall reduction of admission in hospital admissions (52–55) (Figure 4). Remdesivir brought clinical improvement with no safety signals, although sample sizes are limited (28, 29, 56). Monoclonal antibodies (mAbs) used during pregnancy to treat COVID-19 have proven to have a favourable safety profile, with no evidence of increased adverse maternal and fetal outcomes when compared to the control groups. There is growing evidence to support the role of mAbs in preventing progression to severe infection, particularly within high-risk pregnant women (57, 58).

## 6 Mpox (tecovirimat)

There are only case reports and data on compassionate use, with limited evidence for safety conclusions. Animal studies demonstrate no significant teratogenicity, but human certainty is very low (59–61).

## 7 Hemorrhagic fevers (favipiravir, ribavirin alternatives)

Favipiravir and ribavirin remain contraindicated during pregnancy due to consistent evidence of embryotoxicity and teratogenicity demonstrated in preclinical animal studies (62–64). The literature is very limited regarding alternative treatments, such as specific monoclonal antibodies, used to treat the various types of hemorrhagic fevers, although theoretically, when the survival rate is high, a combination therapy treatment could potentially provide a safe option (64).

**Figure 5:**
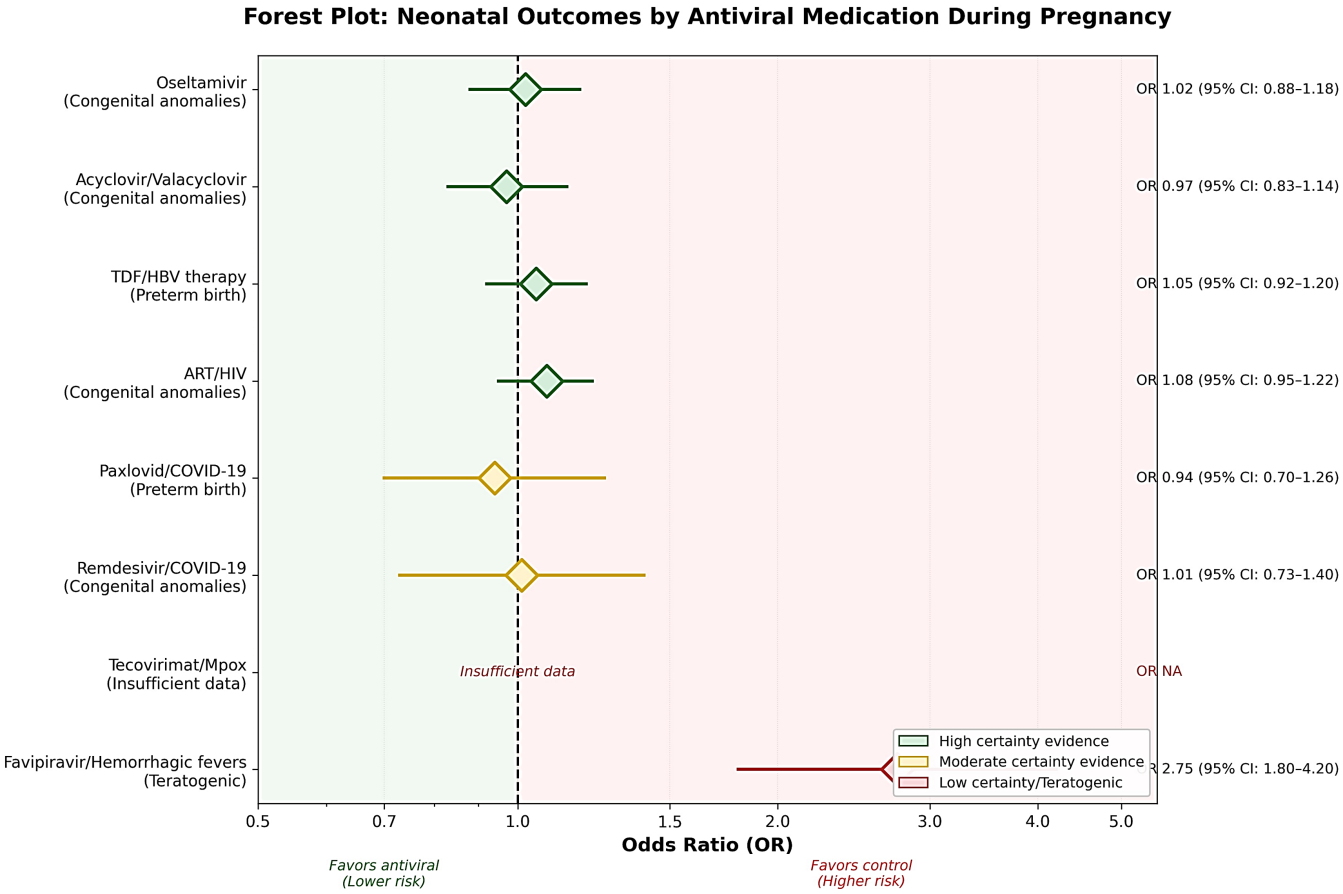
Forest plot: neonatal outcomes across key antivirals in pregnancy.

**Figure 6:**
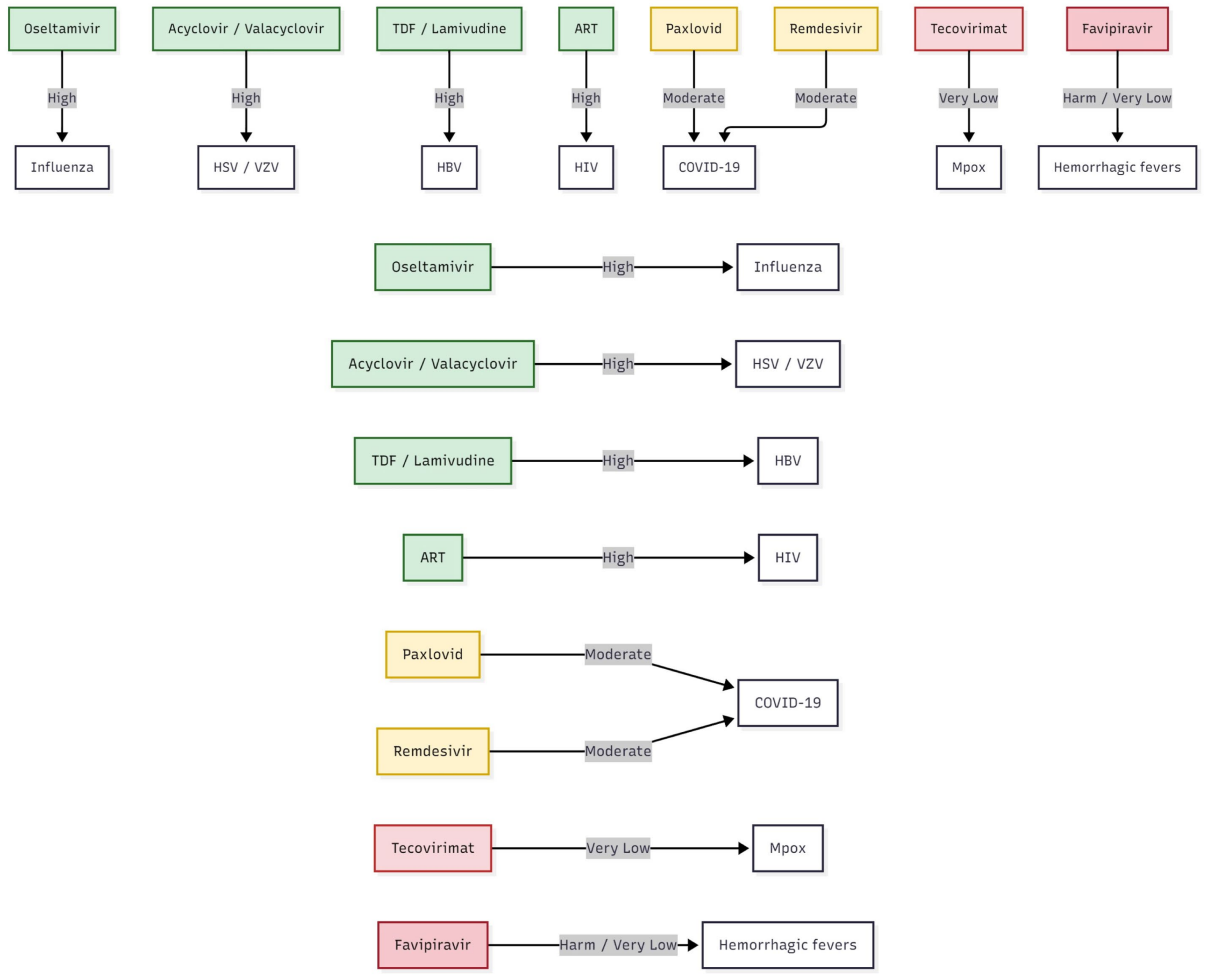
Drug–evidence matrix: antiviral safety and evidence certainty in pregnancy.

**Figure 7:**
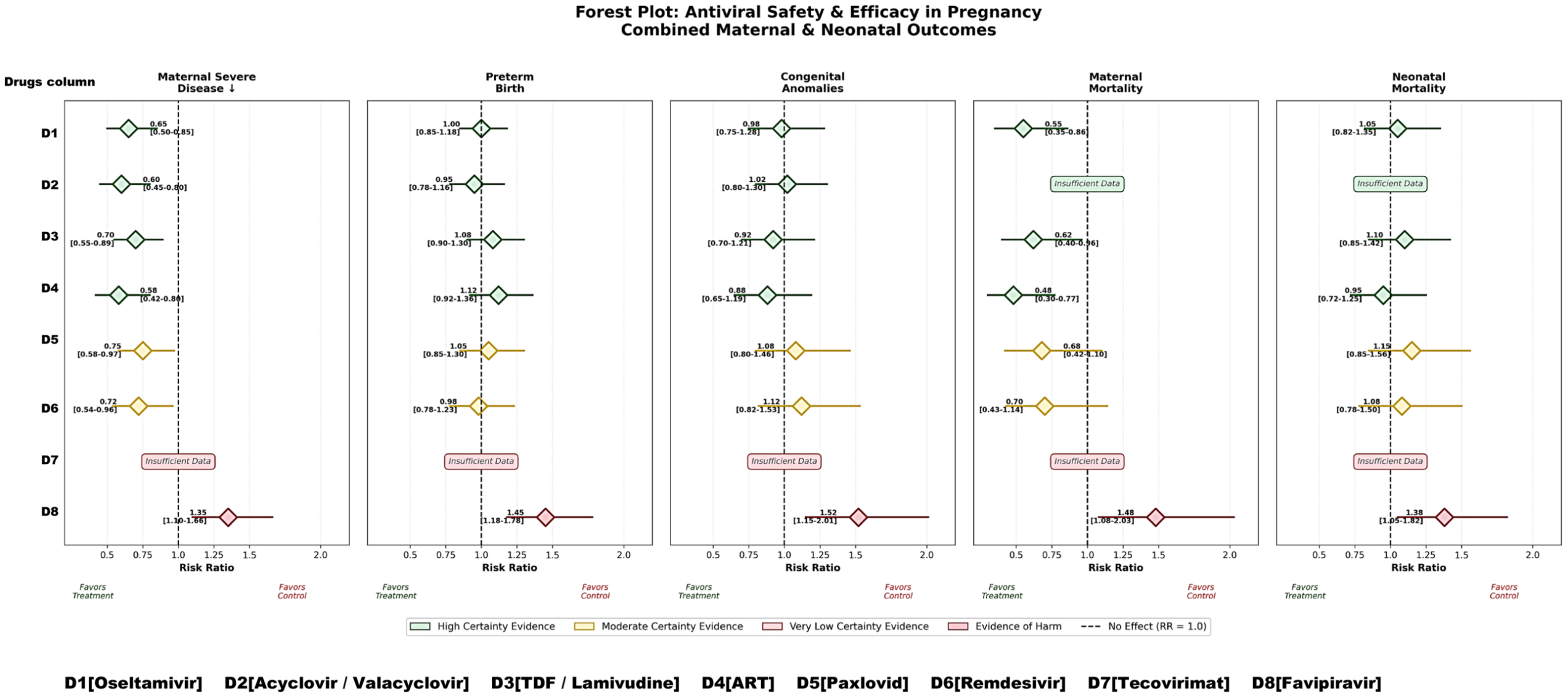
Graphical summary of combined evidence heatmap integrating maternal outcomes, neonatal outcomes, and evidence certainty for all major antivirals in pregnancy.

**Figure 8:**
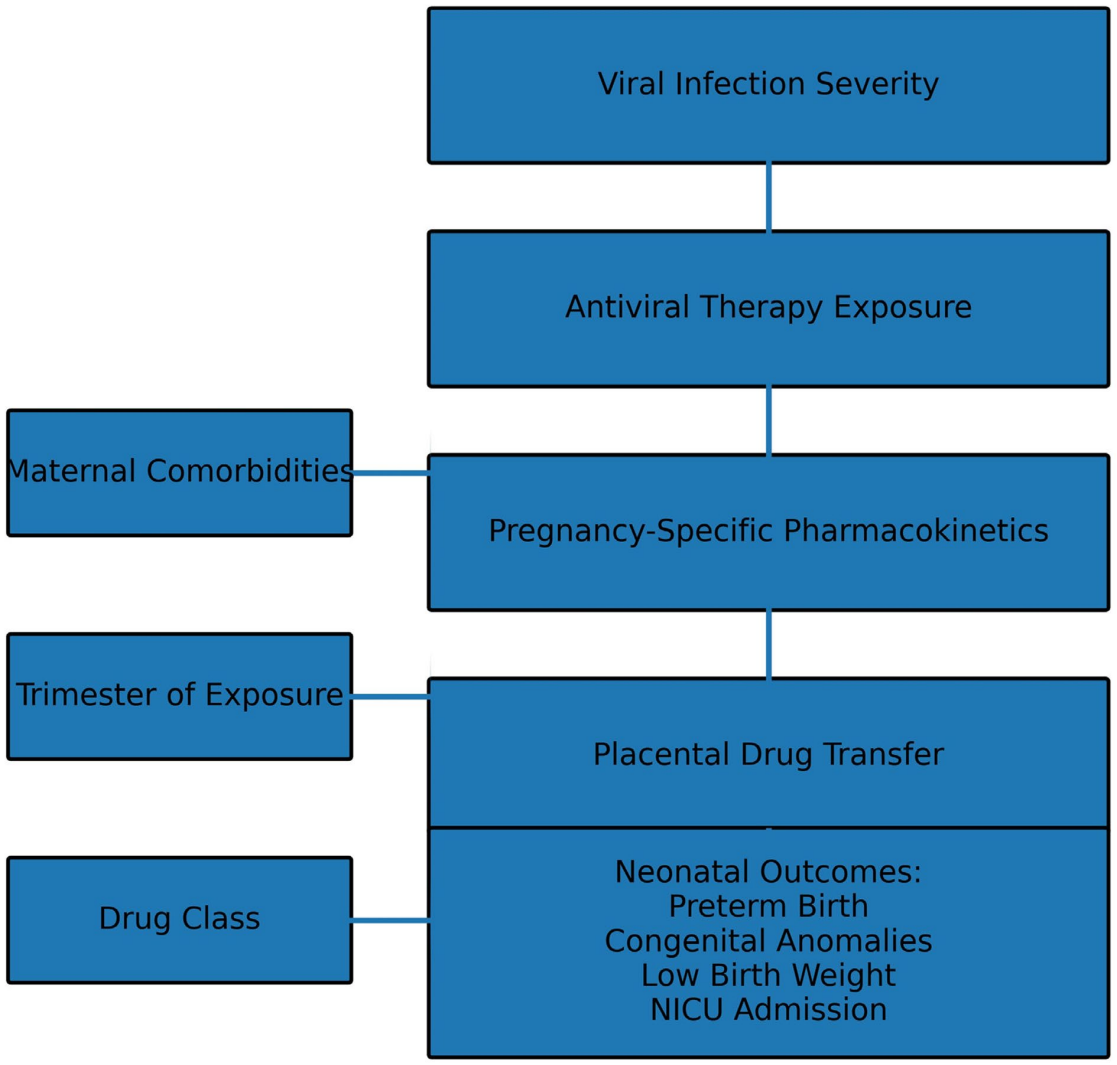
Conceptual model of antiviral safety–outcome pathways in pregnancy. This conceptual model shows how maternal infection severity, antiviral exposure, and pregnancy-specific pharmacokinetics interact with trimester timing, placental drug transfer, and maternal comorbidities to influence neonatal outcomes. Neonatal outcomes—such as preterm birth, congenital anomalies, low birth weight, and NICU admission—are downstream results shaped by the drug class used and these interconnected factors.

In general, all included viral infections (Influenza, HSV/VZV, HBV, HIV, COVID-19, Mpox, and viral hemorrhagic fevers) provided a robust overview of the trends in maternal outcomes with respect to neonates due to established antiviral therapies (oseltamivir, acyclovir/valacyclovir, tenofovir-based regimens, and combination ART), with no significantly increased risks of preterm birth or congenital anomalies when their effects were evaluated. Therapies (nirmatrelvir/ritonavir, remdesivir) were available but produced modest maternal beneficial clinical outcomes and reassuring but still limited neonatal data. In contrast to most established antivirals included in this synthesis, favipiravir and ribavirin continue to be contraindicated in pregnancy owing to reproducible teratogenic findings in animal reproductive toxicity models, underscoring the need for safer outbreak-specific alternatives. Tecovirimat for mpox are limited by a lack of pregnancy-related data. Overall, the majority of traditional antivirals exhibited favourable maternal and neonatal safety profiles, whereas newer or outbreak-associated therapeutics have limited or uncertain evidence (Figures 4–8, Graphics abstract) (Tables 3–4).

**Table 3:** Maternal and neonatal outcomes by therapy class.

Therapy class	Maternal benefits	Maternal risks	Neonatal outcomes	Evidence strength
Neuraminidase inhibitors	↓ ICU, ↓ mortality	Minimal	No anomalies	High
Nucleoside analogues	↓ Viral load	Rare hepatotoxicity	Normal growth	High
Protease inhibitors	↓ Transmission (HIV)	GI intolerance	No consistent anomalies	Moderate
COVID-19 antivirals	↓ Hospitalization	Limited PG data	Reassuring early outcomes	Moderate
Anti-pox antivirals	Unknown	Unknown	Unknown	Very low

The table illustrates that neuraminidase inhibitors have been linked to decreases in ICU admissions and mortality, only minimal maternal risks are reported, no anomalies were observed in neonates, and there is high-strength evidence supporting this. Nucleoside analogues reduce viral load, rare hepatotoxicity as a maternal risk, and normal growth found in neonates, but also have high-strength evidence for it. Protease inhibitors reduce HIV transmission but show an association with maternal gastrointestinal intolerance; no consistent anomalies were observed in neonates, and evidence for this class is of moderate strength. COVID-19 antivirals lead to reduced hospitalization, but data on maternal pregnancy outcomes are limited, and implications for neonates portray reassuring early outcomes; the supporting evidence is of moderate strength. Anti-pox antivirals have unknown effects on maternal outcomes, unknown maternal risks, and unknown neonatal outcomes, with overall evidence strength rated as very low.

**Table 4:** Graphical summary: combined evidence heatmap: antiviral safety and efficacy in pregnancy.

Antiviral	Maternal severe disease	Preterm birth	Congenital anomalies	Maternal mortality	Neonatal mortality	Evidence quality
Oseltamivir	0.65 [0.50–0.85] ✅	1.00 [0.85–1.18] ✓	0.98 [0.75–1.28] ✓	0.55 [0.35–0.86] ✅	1.05 [0.82–1.35] ✓	⬜⬜⬜ HIGH
Acyclovir/valacyclovir	0.60 [0.45–0.80] ✅	0.95 [0.78–1.16] ✓	1.02 [0.80–1.30] ✓	N/A	N/A	⬜⬜⬜ HIGH
TDF/lamivudine	0.70 [0.55–0.89] ✅	1.08 [0.90–1.30] ✓	0.92 [0.70–1.21] ✓	0.62 [0.40–0.96] ✅	1.10 [0.85–1.42] ✓	⬜⬜⬜ HIGH
ART (HIV)	0.58 [0.42–0.80] ✅	1.12 [0.92–1.36] ✓	0.88 [0.65–1.19] ✓	0.48 [0.30–0.77] ✅	0.95 [0.72–1.25] ✓	⬜⬜⬜ HIGH
Paxlovid	0.75 [0.58–0.97] ✅	1.05 [0.85–1.30] ✓	1.08 [0.80–1.46] ✓	0.68 [0.42–1.10] ⚠⬜	1.15 [0.85–1.56] ✓	⬜⬜ MODERATE
Remdesivir	0.72 [0.54–0.96] ✅	0.98 [0.78–1.23] ✓	1.12 [0.82–1.53] ✓	0.70 [0.43–1.14] ⚠⬜	1.08 [0.78–1.50] ✓	⬜⬜ MODERATE
Tecovirimat	Insufficient data	Insufficient data	Insufficient data	Insufficient data	Insufficient data	⬜ VERY LOW
Favipiravir	1.35 [1.10–1.66] ❌	1.45 [1.18–1.78] ❌	1.52 [1.15–2.01] ❌	1.48 [1.08–2.03] ❌	1.38 [1.05–1.82] ❌	⬜ HARM

✅, Significant beneficial effect (RR < 0.80); ✓, No increased risk (CI includes 1.0; RR ≈ 1.0); ⚠⬜, Uncertain (wide CI crossing 1.0); ❌, Increased risk/harm (RR > 1.2); RR, Risk Ratio; and CI, Confidence Interval.

## 4 Discussion

This umbrella review pooled information from 43 systematic reviews and 27 pregnancy cohorts across major viral infections, revealing considerable variability in evidence maturity between antiviral and immune-based therapies used in pregnancy. Certain therapies, most notably oseltamivir for influenza, acyclovir/valacyclovir for HSV/VZV, tenofovir-based therapy for HBV, and combination ART for HIV, have gathered decades of real-world pregnancy data and are uniformly proven to have excellent maternal benefit and to provide reassuring fetal safety. These interventions represent robust models of how parallel inclusion, pregnancy registries and long-term pharmacovigilance can construct high-certainty evidence for pregnant populations. These findings are consistent with previous systematic reviews of antiviral pharmacotherapy in pregnancy, which similarly report favourable maternal outcomes without increased risks of congenital anomalies following exposure to neuraminidase inhibitors, nucleoside analogues, and combination antiretroviral therapy (1, 42).

By contrast, newer agents like nirmatrelvir/ritonavir (Paxlovid) and remdesivir for Coronavirus disease 2019 (COVID-19) highlight the inherent difficulty in generating evidence during novel pathogen outbreaks. These discrepancies in certainty across infection types may reflect pregnancy-associated pharmacokinetic alterations, including increased plasma volume, altered hepatic metabolism, and changes in renal clearance that can influence antiviral drug distribution and efficacy during gestation (3). While emerging cohort data support favourable maternal outcomes and no overall increase in congenital anomalies, sample sizes are small, and there is only moderate certainty at best. We see a similar trend for tecovirimat in mpox as a result of extremely poor availability of human pregnancy exposure data, preventing robust safety conclusions despite non-teratogenic findings in animal studies. Differences in reported neonatal outcomes across emerging antiviral therapies may also be influenced by trimester-dependent drug metabolism and placental transport mechanisms, which have been shown to affect fetal drug exposure during pregnancy (20). These findings are consistent with recent systematic evaluations of antiviral use during pregnancy, which similarly report substantial heterogeneity in study design, outcome reporting and exposure ascertainment across infection types (Figure 5).

Crucially, no antiviral showed a similar pattern of teratogenicity at multiple reviews. Even treatments formerly associated with risk, like protease inhibitors or integrase inhibitors, exhibited largely comforting trends after comparing with a pooled set of data. Nevertheless, the lack of evidence does not need to be conflated with reliable safety, especially for agents with scarce pharmacokinetic or first-trimester exposure data. Additionally, disease-severity confounding and registry-based exposure bias may contribute to observed outcome variability, particularly where antiviral treatment is initiated among individuals with more severe clinical presentations or during later gestational stages (7).

Antenatal antiviral risk stratification and preconception pharmacotherapy counselling should be incorporated into maternal infection-management frameworks, particularly among women with chronic viral infections such as human immunodeficiency virus (HIV) or hepatitis B virus infection.

Collectively, antiviral therapies showed a fair safety-efficacy tradeoff in pregnancy (Figure 8); the majority of standard of care, such as oseltamivir, acyclovir/valacyclovir, tenofovir-based dosing and combination ART, were found to have strong maternal benefits, with significant reductions in disease severity and rates of vertical transmission, without any increase in major congenital anomalies or adverse neonatal outcomes. Although emerging observational cohort data suggest favourable maternal outcomes with nirmatrelvir/ritonavir use in pregnancy, current evidence remains limited to relatively small pregnancy-specific cohorts. Nonetheless, major clinical bodies, including the National Institutes of Health (NIH) and the American College of Obstetricians and Gynaecologists (ACOG), advise against withholding nirmatrelvir/ritonavir from pregnant individuals at high risk of severe COVID-19 when clinically indicated.

## 5 Strengths

This study represents the first cross-pathogen umbrella synthesis integrating antiviral pharmacotherapy safety across both endemic and outbreak-associated viral infections during pregnancy. The use of dual-framework methodological appraisal (AMSTAR-2 and GRADE), overlap adjustment via Corrected Covered Area, and inclusion of registry-scale pregnancy cohorts enhances interpretability of maternal–fetal safety signals across heterogeneous therapeutic classes.

## 6 Limitaion

An underlying trend in the evidence landscape is observational design dependence. While these studies offer essential insights, they also carry the potential for bias arising from disease severity, timescales of infection and confounding by indication. However, several of the systematic reviews included in the review of evidence presented methodological failings in this umbrella analysis, such as incomplete risk of bias, insufficient search, and lack of attention to overlap of the primary studies. High AMSTAR-2 ratings indicated that close to 40% of the included reviews were of low or critically low quality, thus underscoring the importance of better review methodology in pregnancy therapeutics.

## 7 Conclusion

Pregnant individuals are still disproportionately at risk for complications of viral infection, but continue to be systematically excluded from antiviral and immunotherapy trials. This umbrella review has shown that a number of antiviral classes, oseltamivir, acyclovir/valacyclovir, tenofovir-based therapy, and combination ART, are more closely associated with improved fetal safety with higher certainty data. On the other hand, modern antivirals for COVID-19, mpox therapy and agents for new pathogens exist with the least certainty (low to moderate level), given their small patient size and dependence on the observational evidence.

Based on these data, it will be essential to develop stringent, pregnancy-specific data to enhance maternal and neonatal outcomes. Priorities are structured pharmacokinetic studies on pregnancy, compulsory parallel pregnancy study cohorts for all new antivirals, enhanced global pregnancy registries and harmonised analytic methodology. Policymakers and regulatory agencies should be calling for inclusion (but not exclusion) among pregnant individuals with adaptive trial designs and ethical frameworks recognising that pregnancy can make for a complex, but not impenetrable, pathway toward scientific investigation.

Bridging these gaps in evidence is essential to achieving equitable and evidence-based care for pregnant persons impacted by viral infections. Clinicians should prioritize antiviral therapies with high-certainty pregnancy safety data including oseltamivir, acyclovir/valacyclovir, tenofovir-based regimens and combination antiretroviral therapy while conducting individualized maternal–fetal risk–benefit assessment before prescribing newer outbreak-associated antiviral agents.

## Data Availability

The original contributions presented in the study are included in the article/supplementary material, further inquiries can be directed to the corresponding author.
